# Comparative study between the excision-ligation and autoligation of vas deferens technique for teaser rams preparation

**DOI:** 10.14202/vetworld.2019.901-908

**Published:** 2019-06-27

**Authors:** Dhurgham Hameed Al Haideri, Hussein Kareem Ibraheim, Falah Baiee

**Affiliations:** Department of Clinical Science, Faculty of Veterinary Medicine, University of Kufa, 54003 Kufa, Najaf, Iraq

**Keywords:** ram infertility, testes, vas occlusion, vasectomy techniques

## Abstract

**Aim:**

The present study was designed to demonstrate the autoligation (AL) of vas deferens and the excision-ligation (EL) technique to generate vasectomized rams to reduce the complications, operative time, and price of the vasectomy techniques.

**Materials and Methods:**

A total of 12 healthy and mature Iraqi Awassi rams were used, which divided into two groups, six rams for each one. The former group was performed the EL technique while the latter group, the AL of vas deferens technique was used.

**Results:**

The results of the present study found that both techniques were same with the reproductive efficient examinations that mean the two techniques had same ability to close the male genital passage for teaser rams preparation. However, the methods were different with the histopathological changes, operation time, prices, and complications, which were minor in the AL of vas deferens compared with the EL technique.

**Conclusion:**

The AL technique of vas deferens to prepare teaser animal is recommended over the EL technique due to different aspects such as cost, fewer complications, and active teaser for a long period are the main aspects of AL technique.

## Introduction

Improving the reproductive quality of the livestock industry is one of the main objectives of veterinarians [1]. Basically, fixed time artificial insemination [2] and estrous synchronization followed by superovulation [3] are used top quality semen that preserved as a liquid [3] or frozen form [4]. However, sometimes, small ruminants tend to silent heat [5], so these techniques might need to determine the estrous using estrous detector. It is well known that sheep is first domesticated at Mesopotamia Area in the Motherland of the Euphrates and Tigris rivers [6]. Among the approximately 1400 sheep breeds are known [7], the fat-tailed Awassi breed has attracted particular attention not only because it is the most numerous breed in the Middle East countries but also due to its high contribution to milk and meat production in global sheep production system [8]. Awassi sheep have very desirable characteristics as far as endurance to nutritional fluctuations, resistance to diseases and parasites, tolerance to extreme temperatures beside its high milk producing, and growth abilities [8]. Even though Awassi sheep is a photoperiod seasonal breeder, it can breed around the year when the feed and right management are provided [9]. Therefore, vasectomized ram could help farmers or veterinarians to detect the estrous in ewes.

A vasectomy is a surgical technique that used to excise partial part of the vas deferens. The testis continues to secrete the androgen hormones so that the male will expose behave like a normal male [10]. There are many methods that can be used for estrus phase detector in female and the most common ones are the surgically modified males. These methods depend on males have the ability to mate the female without ejaculation. Vasectomized males are modified surgically so retain natural sexual desire may occur, but this method is occlusion of the sperms’ passage [11]. The first and oldest method was excision-ligation (EL) of vas deferens [12]. It was the common method that utilized worldwide, due to give ensure occluded of the male genital passages [13]. Then after, the EL technique was modified by changing the side of incision and named by the midscrotal vasectomy [14].

Thermal cautery of the lumen of vas deferens [15], and it considers economic technique [16]. The Intravas device is a fast and easy method [17]. Furthermore, the classic method was modified with no-scalpel vasectomy to reduce the prices, complication, and operative time [18].

The present study was designed to demonstrate the autoligation (AL) of vas deferens and the EL technique to generate vasectomized rams to reduce the complications, operative time, and price of the vasectomy techniques.

## Materials and Methods

### Ethical approval

The experimental design was approved by the Institutional Animal Care and Use Committee (UOK10/2016), University of Kufa, Iraq.

### Animals

The current study was inducted on 12 Iraqi Awassi rams. Rams aged from 3 to 4 years old and the weight of the rams was between 45 and 52 kg. Rams were kept in a separate crate in the sheep farm at the Faculty of Veterinary Medicine, University of Kufa, Najaf, Iraq. The animals were fed with grass, barley hay, and bran flour. Tap water and blocks of minerals were provided *ad* *libitum* to the animals.

### Preparation of animals before surgical operation

Rams were divided into two equal groups, six rams for each group. Two methods of vasectomy were performed under local anesthesia. All males fasted for overnight before the day of surgery. The skin area at the base of the cranial aspect of the scrotum was clipped, shaved, and prepared aseptically. Each ram was sedated using xylazine 2% (Hoge Mauw/Belgium) 0.2 mg/kg b.w., lignocaine 2% (Norbrook UK) was infiltrated subcutaneously and intramuscularly. The first group was performed EL, while the second group was used AL of vas deferens by the followed techniques:

### EL of vas deferens technique

**Figure-1:**
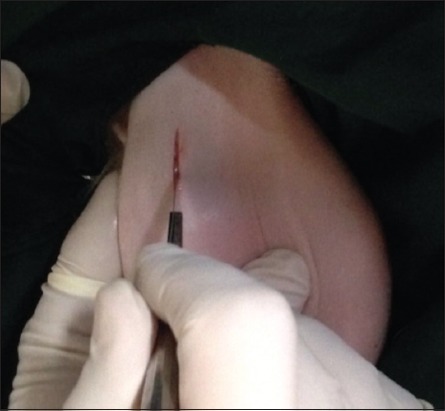
Incision created by scalpel.

**Figure-2:**
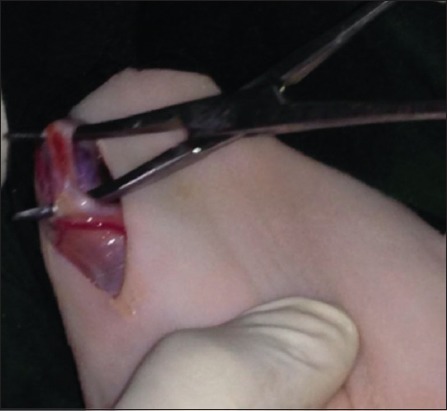
Vas deferens isolated and exited through the incision.

**Figure-3:**
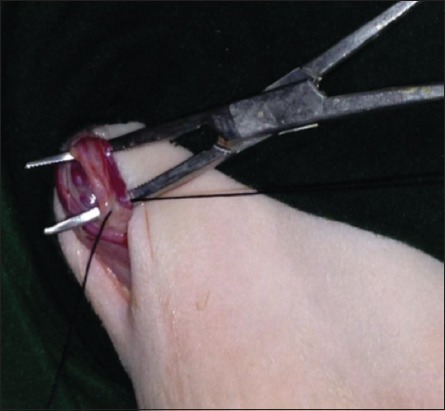
Vas deferens ligation.

**Figure-4:**
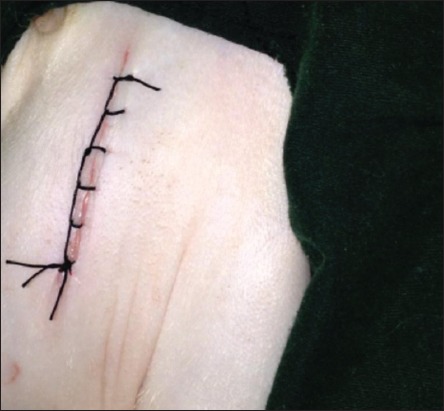
Skin sutured by the ford interlocking pattern.

The skin was incised about 2-3 cm (Figure-1); it was performed at the base of the scrotum through scalpel, and the vas deferens was detected by palpation. The vas deferens was isolated and exited through the incision (Figure-2). A 1-2 cm length excised and ligated the two ends with absorbable suture material (Chromic Catgut/Sumbow; number 0; Figure-3), to remove of a small piece between ligations. This procedure was applied for both testes. The incision of the skin of scrotum was sutured by ford interlocking pattern with non-absorbable suture material (Silk/Sumbow; number 2; Figure-4). The animals treated by the course of antibiotic (Penstrep/UK) for 5 days [19].

### AL of vas deferens technique

**Figure-5:**
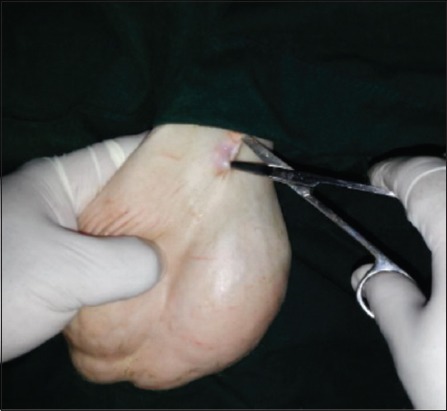
Incision created by sharped scissor.

**Figure-6:**
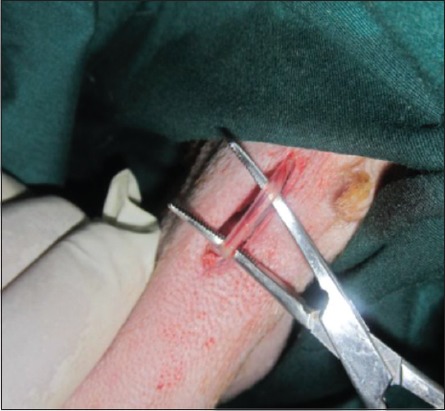
Vas deferens isolated and exited through the incision.

**Figure-7:**
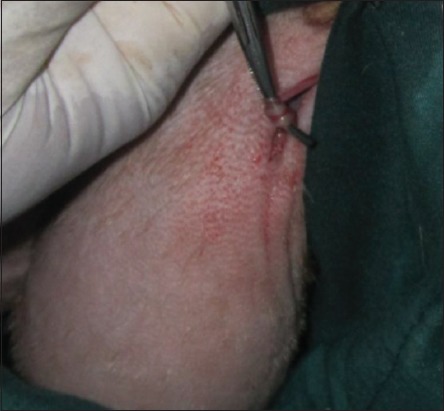
Vas deferens tortured around curved forceps.

**Figure-8:**
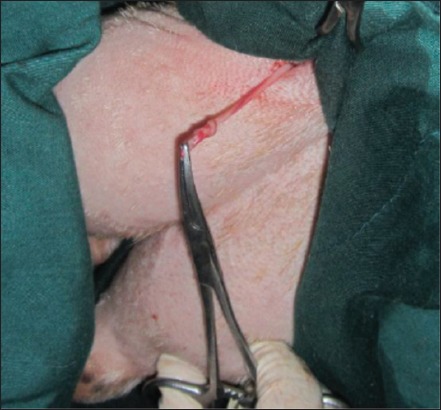
Vas deferens pulled through torsion to create autoligation.

The base of scrotum was incised with a sharped scissor (1-2 cm; Figure-5). The vas deferens was touch by the left index finger externally. The vas deferens was lifted from the spermatic cord structures with small curved forceps throughout the small incision (Figure-6). It was tortured around forceps (Figure-7), inverted to another side to grab the abdominal part of vasa after excised it, this end was passed and pulled through the torsion part to perform the vasa AL (Figure-8). The ligated vas deferens rebounded into the spermatic cord. This procedure was applied for both testes. The incisions covered with sterile gauze. This technique was done according to Ibrahiem [20] with some modifications to reduce hemorrhage, accelerate healing, and reduce cost. A course of antibiotic therapy was given for 5 days. Furthermore, the operation time of both methods was recorded.

### Macropathological and micropathological examination

Macropathological examination was performed on the checked testicular structures of castrated rams in the 30 days post-operation. Furthermore, the micropathological examination was estimated by fixed the 1 cm^3^ samples of the testicular parenchyma, epididymis, and vas deferens with buffered formalin after the 30 days post-surgery for two techniques.

### Reproductive performance

The reproductive efficient of rams was evaluated by measuring of a sexual desire time; for this purpose, rams were individually liable to an estrous ewe for 15 min and three sexual behavior tests were performed. Testing of rams was evaluated morning between 09.00 and 10.00 am. Ewes were induced into estrous vaginal sponges for 12 days; then, after pregnant mare serum gonadotropin (PMSG) was injected intramuscularly [21]. Sexual behavior features of rams were documented as described by Karaca *et* *al*. [22] which are Flehmen response, anogenital sniffing, and mounting duration (minutes). Furthermore, electroejaculator technique, according to Baiee *et* *al*. [23], was conducted to collect semen from rams to determine the volume of semen and concentration of spermatozoa. The reproductive performance parameters were examined for four periods after the surgical operation (1, 2, 3, and 4 weeks) and compared with the control (0), which was taken before the operation.

### Statistical analysis

Data were analyzed and expressed as means and standard error (M±SE). Statistical comparisons between groups were performed using software SPSS version 18 (IBM, USA) with the one-way analysis of variance. Least significant difference was applied to compare between means.

## Results

The data in Table-1 showed that significant differences (p<0.05) in the sexual desire time at 1 and 2 weeks after the surgical operation then return back to the normal levels at 3 and 4 weeks, the data were observed in both techniques. Furthermore, in Table-2, the semen volume recorded a significant decrease at weeks (1, 2, 3, and 4) in EL and AL techniques that compared to the control group (week 0).

**Table-1:** The levels of sexual desire time (minutes) of rams before and after surgical operation.

Method	Periods (weeks)				

	0	1	2	3	4
EL	2.39±0.22^a^	12.7±0.84^b^	4.91±0.4^c^	2.49±0.17	2.62±0.19^a^
AL	2.19±0.16^a^	13.39±0.54^b^	5.07±0.31^c^	2.54±0.19^a^	2.61±0.19^a^

Values with different superscript differ at p<0.05, LSD test. LSD=Least significant difference. EL=Excision-ligation, AL=Autoligation

**Table-2:** The semen volume and concentration of spermatozoa of rams before and after surgical operation.

Period(weeks)	Semen volume/mL		Concentration (10^8^/ml)	
	
	EL	AL	EL	AL
0	1.29±0.56^a^	1.36±0.47^a^	2.53±0.25^a^	2.8±0.21^a^
1	0. 36±0.1^b^	0.56±0.14^b^	0.93±0.10^b^	0.95±0.13^b^
2	0. 28±0.04^b^	0.36±0.10^b^	0.42±0.09^c^	0.50±0.09^c^
3	0.20±0.02^b^	0.24±0.04^b^	0.05±0.02^d^	0.06±0.02^d^
4	0.20±0.01^b^	0.24±0.04^b^	0.00±0.00^d^	0.00±0.00^d^

Values with different superscript differ at p<0.05, LSD test. LSD=Least significant difference, EL=Excision-ligation, AL=Autoligation

The present study revealed that there were no significant differences in the concentration of spermatozoa of both techniques before surgery (week 0). However, the concentrations of spermatozoa were reduced significantly after surgery in both techniques for all four periods compared to the control (week 0).

**Figure-9:**
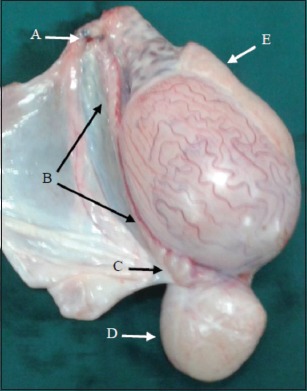
The castrated testis of EL after 30 days: (A) Stitch ligation. (B) Vas deferens. (C) Vas deferens swollen. (D) Severe enlarged epididymal tail. (E) Moderate enlarged epididymal head.

**Figure-10:**
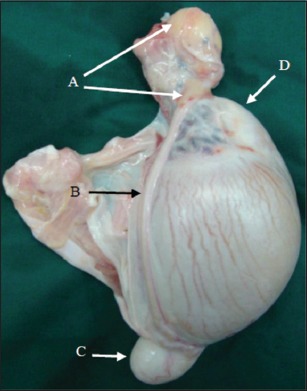
The castrated testis of AL after 30 days: (A) Cyst-like ligated end. (B) Normal size of vas deferens. (C) Moderate enlarged epididymal tail. (D) Moderate enlarged epididymal head.

The post-inspection of castrated testis of EL group showed that there was sever enlargement at the tail of epididymis, mild enlargement at the body and head of epididymis, and swollen in different part of vas deferens. The enlargements were due to accumulation of spermatozoa (Figure-9). In contrast, the AL group observed a major swollen at the ligated end of vas deferens which contained a creamy yellow substance due to the accumulation of semen (Figure-10).

**Figure-11:**
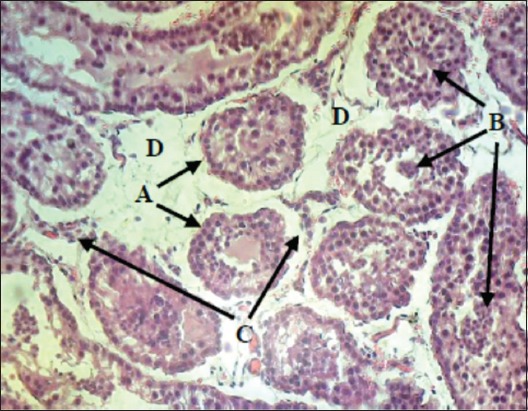
The excision-ligation testicular section after 30 days: (A) Seminiferous tubule shrank and separated from the connective tissue. (B) Sertoli and germinal cells damaging with centrally multinuclear cells accumulation. (C) Leydig cells. (D) Interstitial connective tissue damaging (H and E, 40×).

**Figure-12:**
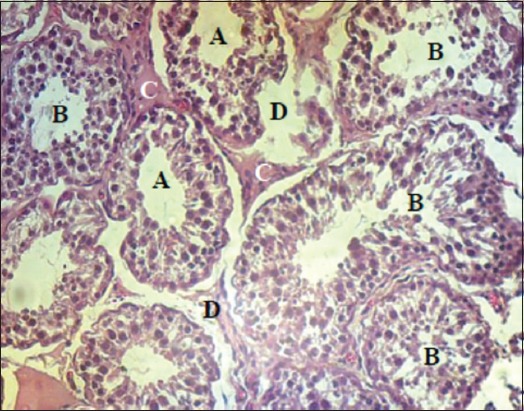
The autoligation testicular section after 30 days: (A) Seminiferous tubule shrank and separated from the connective tissue. (B) Sertoli and germinal cells damaging with multinuclear cells accumulation. (C) Leydig cells. (D) Interstitial connective tissue damaging (H and E, 40×).

Micropathological examination of the EL group showed that most of the seminiferous tubules shrunk and separated from their basal membrane, with accumulation the multinuclear cells at the center of these tubules and separated from the connective tissue. In addition, it was observed the damaging of interstitial connective tissue with the slight proliferation of Leydig cells. However, the Sertoli and germinal cells seemed to be the degenerated (Figure-11). The seminiferous tubules of AL testis suffered slight separation and destruction of the interstitial connective tissue with marked the proliferation of Leydig cells, but the Sertoli and germinal cells were in minimal levels of the degeneration and necrosis (Figure-12).

**Figure-13:**
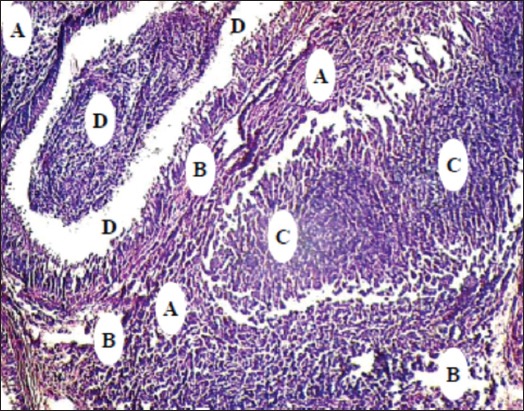
The excision-ligation epididymal tail after 30 days: (A) Connective tissue thickness. (B) Edematous area. (C) Spermatic granulomatous. (D) Epididymal duct dilated and filled by a large sperms mass with epithelial sloughing (H and E, 40×).

**Figure-14:**
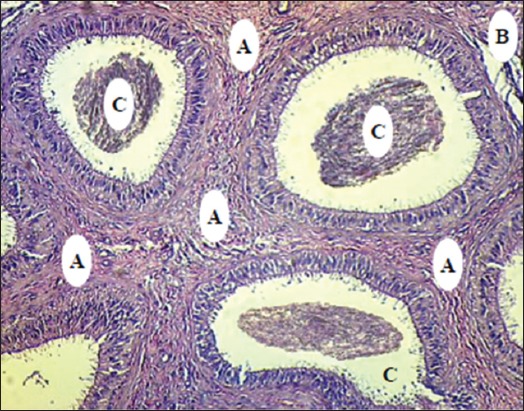
The autoligation epididymal tail after 30 days: (A) Connective tissue thickness. (B) Edematous area. (C) Epididymal duct dilated and filled by a large sperms mass with epithelial sloughing (H and E, 40×).

The epididymal tail of EL technique group was thicker than that of AL technique group by filling with connective tissue and empty areas (edema). In addition, large spermatic granulomatous occurred and the lumen filled with a large spermatozoa mass which caused dilation in the epididymal duct and epithelial sloughing (Figure-13). The epididymal duct of AL technique observed moderate spermatozoa mass accumulation with little dilation; the thickness of connective tissue was mild with few empty areas (Figure-14).

**Figure-15:**
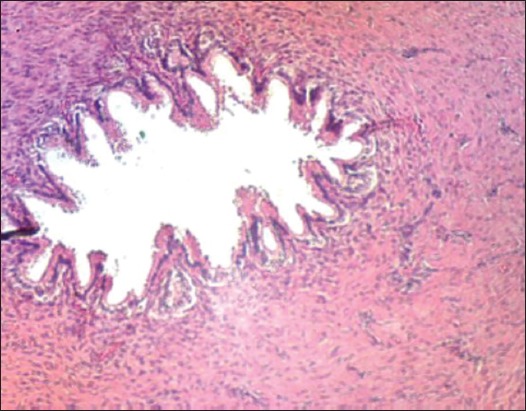
The vas deferens of excision-ligation after 30 days showed damaging and sloughing of vasa by degenerative and necrosis in epithelial layer and connective tissue (H and E, 40×).

**Figure-16:**
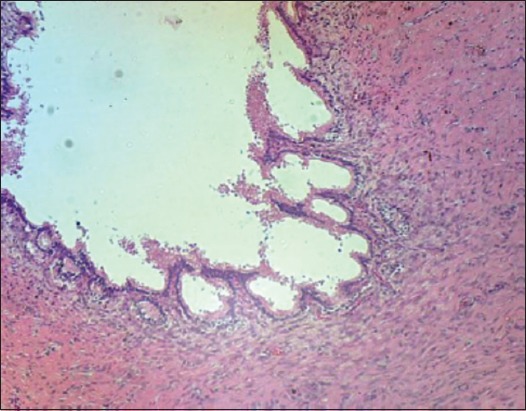
The vas deferens of excision-ligation after 30 days showed severe damaging and sloughing of vasa by degenerative and necrosis in epithelial layer and connective tissue (H and E, 40×).

The vas deferens of EL technique after 30 days post operation showed that there were damaging, sloughing and necrosis in the epithelial layer and in the connective tissue of the vas deferens (Figure-15). The vasa of AL technique seemed the severe degenerative and necrosis in epithelial layer and connective tissue (Figure-16).

The mean of the operation time of EL technique occupied about 11.5 min then after, animals suffered from pain, redness, and swollen for about 7 days as a result of the using of surgical instruments and materials. In contrast, the AL technique continued about 5.75 min due to the steps of the procedure and tools were a little; therefore, that caused the inflammatory symptoms for about 5 days.

## Discussion

Semen volume and concentration of spermatozoa of Iraqi Awassi rams were at normal universal values. Since a handful of studies reported similar values in different breeds of rams [24,25]. They found that the average of semen volume was 1.0 ± 0.2 ml and 1.1 ± 0.1 ml in Bangladeshi indigenous ram and Saint Croix Rams, respectively. Even though the sexual desire time was returned back to the normal value at week 3 post-operation as compared to the control (period 0), teaser cannot be used for estrous phase detection solely after week 4 post-operation. This was due to in week 3 the ejaculation had sperm in both techniques while the spermatozoa disappeared at week 4 post-operation. In fact, numerous authors such as Julanova *et al.*, [11] Barone *et al.*, [15] Sokal *et al.*, [16] Cook *et al.*, [18] Ibrahiem [20] demonstrated different techniques to create vasectomized patients. In the present study, the reproductive efficient examination of both techniques did not establish the significant differences between the EL and AL techniques. However, both of them had the same ability to close the male genital passage and create a teaser of ram [26] that proved ram loss its fertility after 14 days post-EL vasectomy.

The anatomical changes of male genitalia of both techniques were various: In the EL group, the vasa occlusion by the stitch ligation with intact vas deferens below this closure, caused destruction of this duct, as well as leaked the semen into surrounding tissue. These factors created the semen cysts and extraluminal compression at the deferent areas of vas deferens which generated the severe enlargement of epididymal tail and moderate enlargement of the body and head of epididymis due to the earth gravitation and semen movement. Janett *et* *al*. [26] revealed that the vasectomy in Ram caused spermatic granulomatous and distension of adjacent tissue for vas deferens and epididymal duct to form an abscess-like sac filled with spermatozoa and that led to the loss of sexual desire after 4-month post-vasectomy. Batista *et* *al*. [27] found that the epididymis of vasectomized buck was suffered from the formation of irregular multiple foci; it contained doughy materials of the spermatic granuloma after 16 weeks of vasectomy. The testes of AL group suffered from great swollen of vasa ligated end due to the accumulation of spermatozoa and the destruction of this end due to the stretching during AL that created the enlarged end filled with creamy yellow substances. The present observations were in line with the previous study [20]. The enlargement on head, body, and tail of epididymis was moderate and this enlargement occurred to reduce the intraductal pressure which resulted from the production of spermatozoa. Hence, the spermatozoa were stored at these parts forming a cyst structure before the vasa ligated end. Seppan and Krishnaswamy [28] revealed that the vasectomy caused spermatic granulomatous and distension of epididymis in the short term of post-vasectomy. Lee *et* *al*. [29] in the human study demonstrated that the vasectomized patients suffering from the pain syndrome of post-vasectomy due to epididymal distention by semen.

The histopathological changes were various between both techniques in different structures: In the testicular parenchyma, damaging was vigorous in the EL group more than AL group. This damage included the seminiferous tubules and interstitial tissue cells that inhibited the sexual desire. The intraductus pressure caused destruction of the testicular tissues. Al-Maghrebi *et* *al*. [30] found that the vasectomy led to apoptosis of the germinal cells. Moreover, Kong *et al*. [19] showed that the occluded continuation of vas deferens led to the destruction of seminiferous tubules and interstitial cells. This might lead to inhibit the spermatogenesis to reduce and regulate the formation of sperms and therefore reduces intra-ductus pressure. Furthermore, Byrne *et* *al*. [31] cited that the immune system might affect the function of Leydig cells by the action of macrophages. Thus, the endocrine activity, testosterone level might be affected. On the contrary, Ren *et al*. [32] revealed that the testosterone level declined 3 days after vasectomy of male rats and this might due to the interstitial tissue damage and fibrosis formation.

The continued spermatogenesis after vasectomy in both groups led to destroy the male genital ducts that caused formation of spermatic granulomatous in the connective tissue that is surrounding the epididymis and sloughed the epithelial layer of vas deferens and epididymis, these signs were more severity in the epididymis of EL group than AL group as a result of the severe distention, while they were more severity in the vas deferens of AL group than EL group due to the stretching during AL. Flickinger *et* *al*. [33] recorded that the cysts look-like spermatic granulomatous information in the destroyed vas deferens and epididymis of vasectomized Lewis rats that stimulated autoimmunity for the leaked sperms.

The mean period of EL technique was longer compared to the AL technique. This long time was due to the type of technique used. Moreover, EL technique was more expensive than AL technique due to EL technique needs many instruments and materials than AL which is simple and useful. Seaman and Harner-Jay [34] mentioned that there were numeral vasectomized techniques, but the best one was the less cost than others due to a little pricing and its economic value.

The complications of EL technique were more than AL technique as a result of the formerly used suture materials which stimulated the tissue inflammation as a foreign body and increased the inflammatory signs (pain, edema, and redness). Diegelmann and Evans [35] mentioned that the foreign materials, degraded tissue, and microorganisms are work as chemotactic agents to pull the inflammatory cells and elongate the inflammatory phase.

## Conclusion

Both techniques were efficient methods to prepare teaser rams, but the AL technique was better than EL technique in some aspects. The cost of AL technique was cheaper. Moreover, AL technique had fewer complications and can be performed in minimum instruments and materials at the clinic. The vasectomized ram of AL group can be remained active as a teaser male for long period than the EL ram due to a little testicular parenchyma destruction to decrease the intraductal pressure in the AL method.

## Authors’ Contributions

DHA, HKI, and FB participated in the study conception and design. DHA and HKI: acquisition of data. HKI and FB: analysis and interpretation of data. HKI and FB: drafting of the manuscript. All authors critically revised the manuscript for important intellectual content and approved the final manuscript.
